# Molecular Mechanisms of Liver Fibrosis in HIV/HCV Coinfection

**DOI:** 10.3390/ijms15069184

**Published:** 2014-05-26

**Authors:** Claudio M. Mastroianni, Miriam Lichtner, Claudia Mascia, Paola Zuccalà, Vincenzo Vullo

**Affiliations:** 1 Department of Public Health and Infectious Diseases, Sapienza University, Piazzale Aldo Moro 5, 00185 Rome, Italy; E-Mails: miriam.lichtner@uniroma1.it (M.L.); claumascia@tiscali.it (C.M.); paola.zuccala@libero.it (P.Z.); vincenzo.vullo@uniroma1.it (V.V.); 2 Infectious Diseases Unit, Sapienza University, Polo Pontino, SM Goretti Hospital, Via Canova, 04100 Latina, Italy

**Keywords:** HIV, HCV, liver fibrosis, cytokines, microbial translocation, matrix metalloproteinases

## Abstract

Chronic hepatitis C virus (HCV) infection is an important cause of morbidity and mortality in people coinfected with human immunodeficiency virus (HIV). Several studies have shown that HIV infection promotes accelerated HCV hepatic fibrosis progression, even with HIV replication under full antiretroviral control. The pathogenesis of accelerated hepatic fibrosis among HIV/HCV coinfected individuals is complex and multifactorial. The most relevant mechanisms involved include direct viral effects, immune/cytokine dysregulation, altered levels of matrix metalloproteinases and fibrosis biomarkers, increased oxidative stress and hepatocyte apoptosis, HIV-associated gut depletion of CD4 cells, and microbial translocation. In addition, metabolic alterations, heavy alcohol use, as well drug use, may have a potential role in liver disease progression. Understanding the pathophysiology and regulation of liver fibrosis in HIV/HCV co-infection may lead to the development of therapeutic strategies for the management of all patients with ongoing liver disease. In this review, we therefore discuss the evidence and potential molecular mechanisms involved in the accelerated liver fibrosis seen in patients coinfected with HIV and HCV.

## 1. Introduction

Hepatitis C virus (HCV) infection is prevalent among human immunodeficiency virus (HIV)-infected populations, with about 7 million people worldwide being coinfected [1]. The risk of liver-related mortality, predominantly attributed to HCV, has decreased since potent antiretroviral therapy (ART) became available and among the non-AIDS causes of death malignancies are nowadays the leading cause of death. The introduction of ART and the greater understanding of the life cycle of HCV and its interactions with the host have resulted in a reduction of hepatic decompensation and mortality in HIV/HCV coinfected subjects. However, liver-related mortality remains still higher among coinfected individuals compared with those with only HIV or HCV monoinfection [2].

Interestingly, natural history studies have shown that HIV coinfection promotes accelerated HCV-related hepatic fibrosis progression, even with HIV replication under full control by ART [3].

Although complex and multifactorial, the pathogenesis of accelerated hepatic fibrosis among HIV/HCV coinfected individuals is beginning to come to light [4]. HIV alters the natural history of HCV-related liver disease through mechanisms that are independent of its effects on T cell-mediated immunity [5]. The most relevant mechanisms involved include direct viral effects, immune/cytokine dys-regulation, altered levels of matrix metalloproteinases and fibrosis biomarkers, increased oxidative stress and hepatocyte apoptosis, HIV-associated gut depletion of CD4 cells, and microbial translocation. HIV also generates a metabolic pathway that leads to liver toxicity and processes such as steatosis and insulin resistance, which may aggravate liver disease. Finally, the potential role in liver injury of heavy alcohol consumption, as well drug use, should be taken into consideration [3,6].

In this review, we discuss the evidence and potential molecular mechanisms involved in the accelerated liver fibrosis seen in patients coinfected with HIV and HCV.

## 2. Pathophysiology of Liver Fibrosis in Chronic Hepatitis C Virus (HCV) Infection

### 2.1. Background Issues

Liver fibrosis is a reversible and dynamic response to hepatic injury. It can be caused by toxic, metabolic, or viral insult, and occurs when there is an imbalance in extracellular matrix (ECM) protein turnover, *i.e.*, enhanced synthesis and reduced degradation. If, as in chronic HCV infection, the injury is prolonged, inflammation persists and accumulation of ECM proteins exceeds their degradation [7], directly stimulating fibrogenesis [8] and leading to a progressive substitution of liver parenchyma by scar tissue. This in turn causes nodules of regenerating hepatocytes to develop, a feature that defines the progression of fibrosis to cirrhosis [9].

Although recent studies have demonstrated that various types of liver cells are involved in hepatic fibrogenesis, the driving force behind this process is the hepatic stellate cells (HSCs), the primary cell sources of ECM [10,11,12,13]. HSCs are generally quiescent in the hepatic perisinusoidal space [14], but become active in response to chemical stimuli produced by hepatocytes or Kupffer cells following cell injury; these stimuli include reactive oxygen species (ROS), lipid peroxides, growth factors and inflammatory cytokines [10]. The most influential growth factors involved in HSC activation and collagen synthesis are transforming growth factor-β1 (TGF-β1) [15,16] and platelet-derived growth factor (PDGF), which are secreted by hepatocytes and platelets, respectively, during liver injury and inflammation [7,17,18]. Levels of both TGF-β1 and all PDGF isoforms are upregulated during HSC activation, correlating with the development of liver fibrosis and hepatocellular carcinoma (HCC) [19,20,21,22,23,24,25,26]. Once activated, HSCs convert into highly proliferative, myofibroblast-like cells, which produce inflammatory and fibrogenic mediators [7,18]. Myogenic HSCs can differentiate to myofibroblasts, which possess pro-fibrogenic potential; in fact, myofibroblasts actively secrete ECM, including α-smooth-muscle actin (α-SMA) and fibrillar collagens (collagens I and III) [27]. HSCs also produce tissue inhibitors of metalloproteinases (TIMPs), which may reduce ECM degradation through suppression of the matrix metalloproteinase (MMP) activity [28].

### 2.2. Interplay between HCV and Liver Inflammation and Fibrosis

**Figure 1:**
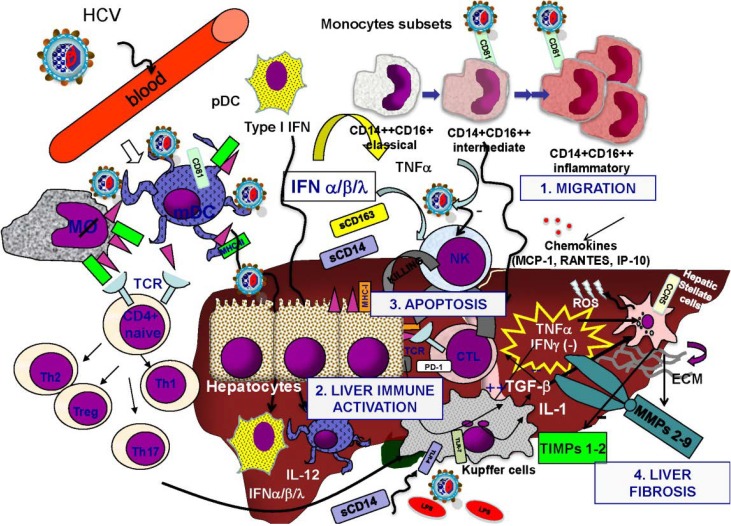
Immunopathogenesis of hepatitis C virus (HCV)-induced liver damage. HCV directlyinteracts with monocytes/macrophage/dendritic cell compartment via CD81 or toll-like receptor (TLR)-7 inducinga pro-inflammatory state and allowing the presentation to naive T and B cells. The increased circulation of chemokines leads to the migration of all these cells into the liver. Natural killer (NK) cells play a central role in control viral replication, but HCV is able to inhibit NK functions. HCV in the liver causes infection of hepatocytes that are a target for the activated effector cells, such as cytotoxic T lymphocytes (CTL), NK, myeloid dendritic cells (mDC) inducing apoptosis. A complex state of chronic immune activation is maintained in the liver and includes also Kupffer cells. In this milieu of pro-inflammatory cytokines (interleukin (IL)-1, tumor necrosis factor (TNF)-α, transforming growth factor (TGF)-β1, IL-12) and oxidative stress (ROS), hepatic stellate cells (HSC) are activated to produce extracellular matrix and to induce a dysregulation of the imbalance between matrix metalloproteinases (MMPs) and tissue inhibitors of matrix metalloproteinases (TIMPs), thus leading to liver fibrosis. Th, T helper; IFN, interferon; sCD, soluble CD; RANTES, regulated on activation normal T cell expressed and secreted; CCR5, *C*–*C* chemokine receptor type 5; ECM, extracellular matrix; LPS, lipopolysaccharide; pDC, plasmacytoid dendritic cells; IP, Interferon-gamma-induced protein; TCR, T-cell receptor.

Chronic HCV is characterized by progressive damage to liver tissue that leads to progressive fibrosis, potentially resulting in cirrhosis, liver failure and HCC [29]. Chronic inflammation is a major contributor to such diseases, and is the basis of HCV-mediated liver damage (Figure 1) [30]. Immunophenotyping of lymphocytes that infiltrate the liver during HCV infection show that they include natural killer (NK), natural killer T (NKT), regulatory T cells, monocytes/macrophages, dendritic cells (DC) and, predominantly, CD4^+^ and CD8^+^ cells, suggesting that the host immune system is involved in the pathogenesis of the resulting liver disease [31,32,33,34,35].

The T-cell response is essential for identification and clearance of HCV, either by cytolysis of virus-infected cells or non-cytolytic clearance via cytokine or chemokine-mediated effects. A greater T-cell response (both virus-specific CD4^+^ and CD8^+^ cells) during acute, rather than chronic, HCV has been reported [36,37,38], and a strong influence of chemokine-chemokine receptor interactions on the recruitment of T cells to sites of inflammation in the liver during chronic HCV infection has been reported [39]. Some genetic studies have found that polymorphisms in the HLA class I and class II molecules on chromosome 6, which are linked to CD8 and CD4 responses, respectively, are associated with spontaneous HCV clearance, thereby confirming the importance of T cells in the elimination of HCV infection [40,41,42,43].

Expression of intrahepatic chemokine ligands and their receptors has been associated with severe HCV-induced liver inflammation [44,45,46]. The release of inflammatory cytokines and chemokines is induced by the crosstalk between HSCs and HCV-infected hepatocytes [47]. It is likely that inflammatory cell activation is triggered by HCV core and NS3 proteins inducing interleukin (IL)-1 receptor-associated kinase (IRAK) activity through toll-like receptor 2 (TLR)-2 [48]. HCV-associated IRAK activation may also contribute to the induction of cytokines and chemokines by HSCs.

The expression of *C*–*C* chemokine receptor type 5 (CCR5) on activated T cells relies upon their recruitment to the liver [49]. Indeed, intrahepatic expression of the ligands for CCR5 (RANTES, MIP-1β, and MIP-1α), which have been linked to a high grade of liver inflammation [50], is elevated in HCV-infected patients. Chronic HCV infection is also known to be associated with increased levels of tumor necrosis factor (TNF)-α in the liver and serum of patients [51,52]. Considering that TNF-α elevation may interfere with insulin signaling [53], this cytokine could be the key molecular link between inflammation, steatosis, and fibrosis in chronic HCV infection. At present, however, we can state that HCV infection induces the generation of inflammatory cytokines and chemokines, potentially leading to the recruitment of inflammatory cells such as cytotoxic T lymphocytes (CTL), neutrophils, monocytes, DCs, and NK cells to the liver, causing liver cell injury and chronic hepatitis [33,34,35].

HCV infection also promotes the activation of macrophages, in particular Kupffer cells, which release ROS and large amounts of proinflammatory and fibrogenic mediators [54,55,56,57], such as TGF-β1. Several studies have demonstrated increased TGF-β1 secretion from HCV-infected cells, feasibly driving HSC activation and hepatic fibrogenesis [58,59,60,61,62]. In addition, both Kupffer cells and activated human HSCs express TLR4—the main target of lipopolysaccharide (LPS), which is released in great amounts during microbial translocation associated with both HCV and HIV infections.

The inflammation and fibrosis of the liver seen during chronic HCV infection is also closely related to the increased apoptosis of damaged hepatocytes. Indeed, a growing body of evidence from both experimental and clinical studies suggests that hepatocyte apoptosis may contribute to liver fibrogenesis by promoting the activation of Kupffer cells and stimulating the fibrogenic action of liver myofibroblasts [63]. Following the uptake of apoptotic bodies [64], Kupffer cells express death ligands such as TNF-α, TRAIL and FasL [65,66,67,68,69,70]. All these death ligands can induce apoptosis in hepatocytes via death-receptor-induced signaling cascades, and thereby aggravate liver injury [71]. In addition, activated myofibroblasts are able to engulf apoptotic bodies and subsequently produce profibrogenic factors such as TGFβ-1 [65].

## 3. Accelerated Liver Fibrosis in Human Immunodeficiency Virus (HIV)/HCV Coinfection

Multiple factors are implicated in the mechanisms of liver fibrosis progression in patients with HIV/HCV coinfection (Table 1).

### 3.1. Direct Effects of HIV

Several epidemiological and clinical findings indicate a direct role of HIV in inducing liver fibrinogenesis [72,73] even in patients with no concomitant viral hepatitis coinfection. In a large North American study in four groups of women (HCV monoinfected, HIV monoinfected, HIV/HCV coinfected and HIV-seronegative/HCV-seronegative women), HIV-RNA plasma levels were associated with increased FIB-4 score in the absence of HBV, HCV, ART or alcohol use [74]. Using transient liver elastography, liver damage has been frequently detected in HIV-monoinfected patients [75,76,77] and correlated with high plasma HIV-RNA levels [78]. In addition, the benefit that ART has on the progression of liver damage (both on biopsies and on clinical events) is well documented [79]. The control of HIV replication by ART dramatically reduces risk of hepatic decompensation and risk of dying from liver disease. In biopsy studies this has been correlated also with less inflammation in the histologies obtained.

**Figure 2:**
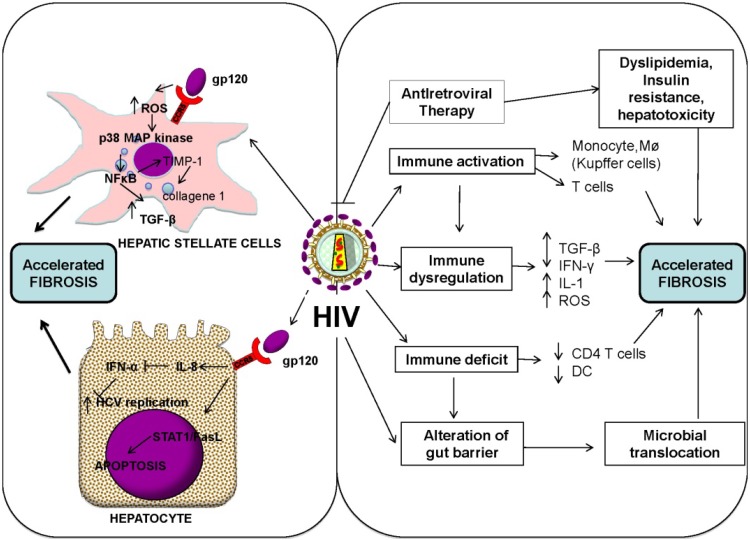
Effect of human immunodeficiency virus (HIV) on liver fibrosis. HIV may induce a direct effect on both hepatic stellate cells and hepatocytes by the interaction between viral proteins (gp 120) and CCR5 (*C*–*C* chemokine receptor type 5) (**left** panel). On the other hand, liver fibrosis could be accelerated by indirect mechanisms, such as HIV immune activation, immune dysfunction and immune deficiency, microbial translocation and toxic effect of antiretroviral drugs (**right** panel).

Therefore, the increased risk of liver disease progression in HIV-monoinfected patients emphasizes the impact of a direct role of HIV in the induction of liver fibrinogenesis (Figure 2) [72,73]. Indeed, hepatocytes (and other resident liver cells) are known to express key HIV co-receptors, including CCR5 and *C*–*X*–*C* chemokine receptor type 4 (CXCR4) [80,81,82,83] and HIV itself has direct cytopathic effect on hepatocytes [73]. A productive HIV infection was demonstrated in hepatocytes, through identification of hepatotropic variants of HIV in autopsied liver tissues [84] and, more recently, in HSC by detection of p24 antigen and HIV-RNA [85].

**Table 1:** Factors associated with liver fibrosis in HIV, HCV and HIV/HCV co-infection.

Markers	HIV	HCV	HCV/HIV
Immune cells			
NK	↓	↓	↓↓
DC	↓↓	↓	↓↓↓
CD4 T cell	↓↓	↓	↓↓↓
Immune activation			
CD4 T cell DR/38+	++	+	+++
CD8 T cell DR/38+	+++	+++	+++
Macrophage (Kupffer cells)	+	++	+++
Cytokines and chemokines			
IP-10	++	++	++++
IL-1β	+	+	++
IFN-γ	↓	↓	↓
TGF-β	+	++	+++
TNF-α	++	+	+++
MIP-1α	↓↓↓	↓↓	↓↓
MIP-1β	↓↓↓	↓	↓↓
RANTES	+++	+	++
Microbial translocation			
sCD14	++	+	+++
LPS	++	+	+++
Fibrosis mediators			
MMP	++	++	+++
TIMPs	++	++	+++
HA	+	++	+++
Apoptosis and ROS			
TRAIL/FAS	++	++	++++
ROS	++	++	+++
Metabolic parameters			
Insulin resistance	++	+	+++
Adiponectin	↓	↓↓	↓↓↓
Resistin	+	++	+++
Leptin	+	++	+++

↓, decrease; ↓↓, moderate decrease; ↓↓↓, marked decrease; +, increase; ++, moderate increase; +++, marked increase; HCV, hepatitis C virus; HIV, human immunodeficiency virus; NK, natural killer; DC, dendritic cell; IP-10, Interferon-gamma-induced protein 10; IL-1β, interleukin-1β; IFN-γ, interferon gamma; TGF-β, transforming growth factor-β; TNF-α, tumor necrosis factor-α; MIP-1α, macrophage inflammatory protein-1α; MIP-1β, macrophage inflammatory protein-1β; RANTES, regulated on activation normal T cell expressed and secreted; sCD14, soluble CD14; LPS, lipopolysaccharide; MMP, matrix metalloproteinase; TIMPs, tissue inhibitors of metalloproteinases; HA, hyaluronic acid; ROS, reactive oxygen species; TRAIL, tumor necrosis factor-related apoptosis inducing ligand.

Both HIV and its envelope protein gp 120 have been shown to induce cell signaling in the liver through interactions with the CCR5 and CXCR4 expressed on the surface of hepatocytes, HSCs and other immune cells [60,85,86,87,88,89], and *in vitro* models have shown that both inactivated HIV and gp 120 enhance HCV viral replication and TGF-β1 expression [57,85,88,90]. This effect of HIV and gp 120 on HCV replication is blocked by antibodies for CCR5 or CXCR4, indicating that CXCR4 or CCR5 co-receptor engagement by HIV is essential for stimulation of HCV replication [57]. The effect of HIV on HCV replication is also blocked by a neutralizing antibody to TGF-β1, one more indicator that HIV itself could directly contribute to hepatic fibrosis and increased HCV replication in a TGF-β1-dependent manner [57].

Transcripts for the chemokine receptors CCR5 and CXCR4 (which bind gp 120) are detectable in human HSCs [89,91,92]. Upon exposure to CCR5-tropic recombinant gp 120, a significant increase in HSC chemotaxis has been observed, as has increased expression of the proinflammatory chemokine monocyte chemoattractant protein-1 (MCP-1), interleukin-6, and tissue inhibitor of metalloproteinase-1 (TIMP-1) [88]. It has therefore been speculated that gp 120 may induce HSC accumulation through direct chemotaxis and secretion of MCP-1 by HSCs themselves, and by activated macrophages, thereby leading to increased liver inflammation and fibrogenesis [85,93,94,95]. Recent experiments using *in vitro* models have also suggested that concurrent exposure of hepatocytes to HCV-E2 protein and HIV gp 120 can directly promote hepatocyte apoptosis through upregulation of FasL expression via the STAT1-dependent pathway. This occurs in a CXCR4-independent manner [81,96] and results in higher viral loads through induction of TGF-1β, a cytokine that dampens the immune response and promotes fibrosis and transformation towards HCC [57,97].

The fibrogenic response by HSCs seems to be mediated by the activation of an autocrine loop involving the chemokine receptor CCR5 [89,98]. Recent experiments in animal models have detected significant reductions in the levels of IL-6, MMP-9, and TGF-β1 when animals were treated with CCR5 antagonists, indicating that these molecules are key regulatory factors of fibrosis through CCR5-signaling interference [99,100,101]. The potential anti-fibrotic effects of maraviroc (MVC), a CCR5-specific HIV-entry inhibitor currently in use in antiretroviral therapy, are currently being investigated in HIV/HCV coinfected patients [102,103]. There is some preliminary evidence that maraviroc, given to such patients to reduce their HIV load, also leads to a reduction in liver stiffness [104]. In addition, treatment with MVC, a CCR5 inhibitor, has also been shown to significantly reduce fibrosis and tumour load in a mouse model of HCC [99].

NALP (NACHT, LRR and PYD-containing protein) 3 inflammasome is a critical step in the proinflammatory signal generation pathway during liver injury, and a possible direct link between the inflammasome pathway in monocytes and HSC and HIV proteins is currently under evaluation. Another likely contributory mechanism to liver fibrosis is the production of reactive oxygen species in HSCs, known to be triggered by both X4-tropic and R5-tropic HIV [87]. Both HIV and HCV act upon hepatocytes and HSCs to stimulate the generation of ROS, which in turn induces p38 mitogen-activated protein kinase (MAPK), c-Jun *N*-terminal kinase (JNK), and extracellular signal-regulated kinases (ERKs), followed by activated nuclear factor kappa (NF-κB). This supports the pro-fibrogenic TGF-β1 genes, which encode collagen and TIMP-1, as well as down-regulating MMP-3 synthesis. Hence both HCV and HIV are directly involved in liver damage by triggering apoptosis and down-regulating antioxidant protective mediators.

### 3.2. HIV-Associated Dysregulation of the Immune Response and Cytokine Network

The quantitative and qualitative impairment of T-cell responses associated with HIV infection may have a negative impact on HCV disease progression. Considering both the critical role of the adaptive immune system in the clearance of HCV and the detrimental effect of HIV infection on T-cells, it is not surprising that HCV persistence in HIV/HCV coinfection is more common than in patients infected with HCV alone. Several lines of evidence indicate that HIV-induced CD4 depletion is independently associated with the severity of liver fibrosis in chronic HCV infection. In particular, a recent study has demonstrated that both HIV-induced loss of CD4^+^ T cells and dysregulated CD4^+^ T cell function lead to a reduction in the anti-fibrotic activity of NK cells, which plausibly results in the accelerated progression of liver fibrosis seen in patients with HIV/HCV co-infection [105]. In addition, a marked intrahepatic CD4 T-cell depletion, associated to an increase in apoptotic lymphocytes in the liver lobule, has been demonstrated in HIV/HCV coinfected patients.

The altered balance between CD4 and CD8 response in HIV infection may be ascribable to a profound dysregulation of the peripheral and intrahepatic cytokine networks, which plays an important role in the accelerated evolution of liver fibrosis (Figure 2). A potential mechanism by which reduced CD4 cell counts promote hepatic fibrosis is a reduction in the secretion of T-helper (Th) 1 cytokine IFN-γ, a well-known anti-fibrotic cytokine. On the other hand, the increased Th2 response seen in HIV infection is associated with secretion of Th2 cytokines (IL-4, IL-5, IL-10, and IL-13), which are known to play a pro-fibrotic role in liver fibrosis [106]. As mentioned above, an important mediator of liver fibrogenesis is the cytokine TGF-β1, which favors activation of hepatic HSC and stimulates ECM production [107]. Significant increases in TGF-β1 expression have been reported in the liver and serum of patients with HIV/HCV coinfection; HIV enhances HCV-related increases in TGF-β1 from hepatocytes, thereby enhancing fibrogenesis.

Another cytokine associated with liver inflammation and immunoregulatory pathways in the liver parenchyma is IP-10 (CXCL10). IP-10 is a CXC chemokine that binds to the CXCR3 immune system receptor present on a number of cells, including monocytes, NK cells and T cells. Various studies have identified elevated IP-10 levels as a negative prognostic indicator for both HCV infection and HIV infection, and it has also been proposed as a negative predictor of response to antiviral therapy in HCV mono-infected and HIV/HCV co-infected patients [108,109]. A high rate of inflammation, and subsequent activation of the endogenous IFN system, as well as significant levels of oxidative damage [110], have been proposed as mechanisms behind IP-10 elevation. It is believed that elevated IP-10 could recruit T cells from the periphery and within the liver, mediating the damage seen in advanced fibrosis [19]. The mechanism by which elevated levels of IP-10 may affect HCV infection in individuals with HIV and HCV coinfection is, however, unknown. Nevertheless, it has recently been suggested that the regulatory protein HIV tat is able to induce IP-10 expression and subsequently increases the replication of HCV and HCV/HIV in coinfected individuals [111].

IL-1 dysregulation may also play a role in maintaining the HSCs in the proliferative state responsible for hepatic fibrogenesis in HCV/HIV coinfection, as shown in a rat model. IL-1β is a central component of the cytokine milieu that accompanies both acute and chronic inflammation and viral disease [112,113]. As a pleiotropic inflammatory factor, IL-1β has also been implicated in promoting tissue pathology and inducing the production of pro-fibrogenic mediators [114,115,116,117]. Inflammasome and IL-1β have also been recognized as potentially key factors in an innate mechanism, alternative to type I interferon, able to recognize nucleic acids (DNA or RNA) and cytoplasmatic viral particles and consequently induce a pro-inflammatory response [118]. Indeed, several viruses are able to activate caspase-1 and induce IL-1β production through inflammasome signaling, highlighting the potential role of inflammasomes in the immune response to viruses [119]. In fact, a recent study has shown that IL-1β production and hepatic inflammation in HCV infection, induced by phagocytic uptake of the virus, are linked and driven through endosomal TLR7 and NLRP3 inflammasome signaling in liver macrophages/Kupffer cells [30]. Moreover, HCV products induce intracellular reactive oxygen species and ion flux, both of which trigger the NLRP3 inflammasome during virus infection. Finally, the HCV p7 protein is a transmembrane cation channel that also drives ion flux, and is thereby also a potential mediator of NLRP3 inflammasome activation during HCV infection [120]. In fact, high levels of IL-1β have been observed in patients from the early stages of HIV infection, suggesting a role of NLRP3 inflammasome in driving the inflammatory response and subsequent liver fibrosis [121].

Finally, recent studies have highlighted the impact of IL-6 levels on liver fibrosis in HIV and HCV coinfected patients. IL-6 is secreted by T cells and macrophages in response to microbial products and other stimuli and it represents a marker of systemic immune activation and liver inflammation [122]. It has been shown that high circulating levels of IL-6 are associated with portal hypertension and decompensated liver cirrhosis in both HIV-infected and -uninfected patients [123]. In a large study on biomarkers of inflammation, it has been reported that higher concentrations of IL-6 in HIV-infected individuals coinfected with HBV and/or HCV were associated with death within 4 years of ART initiation. Moreover, high levels of IL-6 are predictive of failure to antiviral therapy in HIV/HCV coinfected patients [124].

### 3.3. Role of Immune Activation and Microbial Translocation

It has long been recognized that HIV infection is characterized not only by the development of profound immunodeficiency, but also by a marked and persistent cellular immune activation. The central role of immune activation in the pathogenesis and progression of HIV disease has been highlighted by both animal and human studies. It has been demonstrated that the major driving force behind chronic immune activation is the translocation of microbial products from the damaged gastrointestinal (GI) tract to the portal and systemic circulation. During HIV infection, the massive depletion of CD4 T cells in the intestine leads to an alteration of the immune component of the surface of the GI mucosa that can induce greater translocation of LPS [125]. Increased levels of circulating LPS have been shown during HIV infection, suggesting greater immune activation and a consequent progression of the disease, alongside CD4^+^ T cell depletion [126]. LPS also raises levels of macrophage activation marker sCD14, also considered an indicator of inflammation [127]. Interestingly, high plasma levels of sCD14 have been correlated to an increased risk of mortality in HIV-infected patients [128], while LPS has been proposed as a useful biomarker of progression of the disease, regardless of CD4^+^ T cell count or viral load [129]. sCD14 and LPS are undoubtedly important indicators of liver disease progression in HIV/HCV-co-infected persons [129]. LPS has been shown to accelerate liver fibrosis through engagement of TLR-4 on hepatocytes and HSC following membrane binding via LPS-binding protein (LBP) and CD14. These events lead to simultaneous TGF-β1 stimulation through an NF-κB-dependent pathway and Kupffer cell activation, which, in turn, induces the generation of superoxide and the release of pro-inflammatory and pro-fibrogenic cytokines such as TNF-α, IL-1, IL-6, and IL-12, all of which induce liver damage [130].

Microbial translocation in the host with severe liver fibrosis leads to a reduced clearance of bacterial products and increased immune activation (Figure 2) [131]. Consistent with these findings, the high degree of microbial translocation and immune activation observed in patients with both HIV and HCV infection has a great impact on the progression of liver fibrosis. Therapeutic strategies designed to down-regulate the state of immune activation and to counteract microbial translocation may therefore be potentially useful for keeping liver fibrosis in HIV/HCV co-infection under control.

## 4. Dysregulation of Matrix Metalloproteinases and Role of Fibrosis Biomarkers

To reiterate, liver fibrosis is characterized by a pathological accumulation of the extracellular matrix, reflecting the imbalance between enhanced matrix synthesis and reduced breakdown of connective tissue proteins. Extracellular degradation of matrix proteins is regulated by the matrix metalloproteinases (MMPs), a large family of zinc-dependent endopeptidases. Different levels of MMP regulation ensure the constant remodeling of the ECM, including regulation at the gene expression level, cleavage of the pro-enzyme to an active form, and specific inhibition of activated forms by TIMPs [132]. It is feasible that excessive or inappropriate expression of MMPs and/or a reduction in TIMP production could contribute to different pathological conditions, not only infectious diseases and liver fibrosis, including inflammation, wounds, and invasion of cancer cells [11,12,13]. MMP-1/-13 levels do not change in human and animal models of chronic liver disease, but there is a progressive increase in TIMP-1 and TIMP-2 as the fibrosis advances [133]. There is also evidence that activation of HSC in tissue culture models and during fibrotic liver injury *in vivo* is associated with the expression of potent matrix-degrading MMP inhibitors, including TIMP-1 and TIMP-2. With regard to chronic hepatitis C, an association between hepatic fibroproliferation and alterations in circulating and hepatic MMP and TIMP expression has been reported (Figure 1 and Figure 2). HIV/HCV coinfected patients also exhibit a striking increase in circulating TIMP-1 levels, which is more evident in patients with more advanced CD4 depletion. On the other hand, there is no increase in the plasma concentrations of MMP-9, suggesting an imbalance between circulating TIMP-1 and MMP-9 during HIV infection [134]. It is possible that this altered balance may play a potential role in exacerbating the progression of liver fibrosis in HIV patients coinfected with HCV [133].

Cytokines such as TNF-α, TGF-β, IL-1, and IL-6 are involved in the expression pattern of both MMPs and TIMPs. It is conceivable that the cytokine dysregulation documented in HIV contributes to the activation of HSC [135] and the upregulation of TIMPs, which, in turn, promote the progression of hepatic fibrosis by inhibiting matrix degradation. Finally, translocated LPS, which directly correlate with several independent aspects of innate and adaptive immune activation, might also be involved in the regulation of MMP and TIMP expression. Due to its potential role in HIV-associated immunopathology, MMP enzyme activity might constitute a novel therapeutic target in HIV infection [136].

As for fibrosis markers, some studies on patients infected with HCV report that hyaluronic acid (HA) is an accurate indicator of fibrosis and predictor of individual hepatic complications. HA is a biomarker component of the extracellular matrix that is eliminated from the bloodstream by the liver sinusoids [137]. High CD4 counts in patients with HCV/HIV coinfection have been associated with a reduced risk of substantial increases in HA levels and hepatic fibrosis. Indeed, patients with low and stable plasma HA levels have a very low risk of liver disease progression. HA is also a strong predictor of later development of hepatic encephalopathy or liver-related death in HIV-1/HCV coinfection, and a higher annual increase of HA has been reported in patients experiencing liver-related events during follow-up, as compared with those who did not. Hence HA plasma detection, alone or in combination with other non-invasive methods, may be a useful means of monitoring the progression of liver disease and predicting the risk of hepatic complications in HIV/HCV coinfected patients [138].

### 4.1. Role of Metabolic Alterations

**Figure 3:**
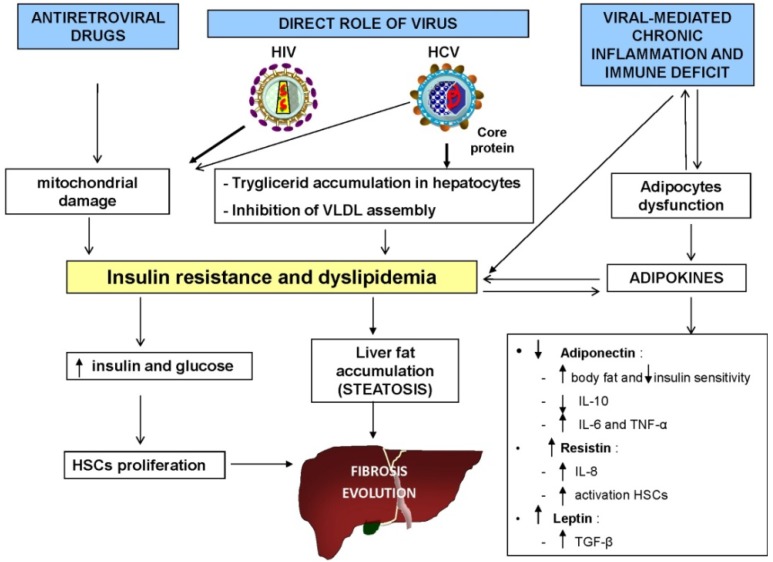
Impact of metabolic alterations and insulin resistance on liver fibrosis progression. Interplay between HIV/HCV coinfection, toxic effects of antiretroviral drugs, and chronic inflammation may play a critical role in the development of hepatic steatosis and fibrosis progression.

Metabolic alterations are common during HIV/HCV coinfection, and play an important role in the genesis of hepatic steatosis, which is closely related to liver fibrosis progression (Figure 3) [139,140,141,142]. These alterations, including dyslipidaemia and insulin resistance (IR), are due to a direct role of the virus, as well as chronic inflammation, deterioration of immunological status, and toxic exposure to antiretroviral therapy. Both HIV itself and the antiretrovirals given to mitigate its effects promote the development of IR and the abnormal deposition of fatty acids in hepatocytes through mitochondrial dysfunction. With regard to antiretroviral drugs and their risk for causing insulin resistance, there are evidence that protease inhibitors, such as indinavir [143] and lopinavir/ritonavir [144], have effects on peripheral lipolysis through inhibition of glucose transporter type 4 (GLUT-4) activity. On the other hand, transcriptase inhibitors (NRTIs), such as zidovudine/lamivudine [145] may affect mitochondrial function which further contribuite to the induction of hepatic insulin resistance in HIV patients receiving ART [146]. Insulin resistance itself may play a key role in the pathogenesis of so-called virus-associated fatty liver disease (VAFLD), which in turn leads to liver steatosis and inflammation [147,148,149,150,151]. Furthermore, high levels of insulin and glucose stimulate HSC proliferation and increase the expression of one of the major factors involved in the progression of fibrosis—connective tissue growth factor (CTGF) [152]. Moreover, there is strong evidence for a central role of mitochondrial dysfunction in the pathophysiology of hepatic steatosis [153]. A recent study also showed that HIV infection has direct effects on mitochondria [154]—HIV and its polypeptides seem to undermine mtDNA and provoke other mtDNA-independent mitochondrial alterations [155].

Emerging evidence suggests that there is a link between immunological and metabolic perturbations. In particular, the increased production of proinflammatory cytokines and perturbance in adipokines (including adiponectin, leptin, resistin, and visfatin) detected in HIV/HCV coinfected patients [141,156] may have an effect on insulin resistance, even promoting metabolic syndrome [139,140]. Another recent study also found that lower levels of adiponectin are associated with advanced hepatic injury [156]. Adiponectin reduces body fat and enhances insulin sensitivity [157], but also reduces inflammation by stimulating secretion of anti-inflammatory cytokines, such as IL-10, inhibiting the release of pro-inflammatory cytokines like IL-6 and TNF-α and blocking nuclear factor κB activation [157].

As previously discussed, HIV/HCV coinfection and the consequent low levels of adiponectin may induce hepatic and systemic inflammation, which could lead to increased expression of resistin and leptin. Several studies have shown that resistin and leptin have a pro-fibrogenic and pro-inflammatory role [158]. Resistin causes expression of pro-inflammatory chemokines and IL-8, induces activation of transcription factor NF-κB, and has pro-inflammatory actions in HSCs [159]. Leptin mediates HSC activation and liver fibrosis through indirect effects on Kupffer cells partially mediated by TGF-β1 [160]. Unsurprisingly, therefore, low serum levels of resistin and leptin could contribute to controlling inflammation and reducing hepatic damage [155].

### 4.2. Role of Alcohol and Drug Use

Most HIV and HCV coinfected individuals, at least in the developed world, are former or active intravenous injection drug users (IVDU) [3]. In addition to heroin, other substances, such as cannabis, amphetamine and cocaine, cause liver injury and fibrosis progression [161]. A study of 272 untreated patients with HCV hepatitis demonstrated that daily cannabis smoking is a risk factor for progression of liver fibrosis [162]. Amphetamines have an extensive hepatic metabolism and the generation of a toxic metabolite may be the cause of hepatic injury [163]. Cocaine can lead to a range of liver abnormalities and its metabolites are involved in oxidative stress and lipid peroxidation in hepatocytes [164,165].

On the other hand, among HIV/HCV coinfected drug users there is a high prevalence of heavy alcohol use [6,166] which plays a potential role in liver disease progression. Indeed, it is known that alcohol is metabolized in the liver and promotes glutathione depletion and lipid peroxidation leading to mitochondrial damage [167,168] and local lymphocyte recruitment [169]. Furthermore, acetaldehyde plays a central role in fibrogenesis by increasing the expression of collagen during HSC activation [170]. Alcohol use may also inhibit the anti-fibrotic action of NK cells [171] and it increases gut permeability and translocation of bacterial products such as LPS [172] which is also involved in fibrogenesis.

## 5. Conclusions

The pathophysiology of accelerated liver fibrosis in patients with HIV and HCV coinfection is a complex and multifactorial process involving viral factors, the immune system, and fibrinogenetic/inflammatory mediators. Hence advances in the understanding of fibrosis pathophysiology and regulation are critical for the development of therapeutic strategies. As new information regarding fibrogenesis comes to light, the key challenge will be translating such advances into the development of anti-fibrotic therapies useful for patients with chronic liver disease, such as that seen in HIV/HCV coinfection.

## References

[1] Kim A.Y., Chung R.T. (2009). Coinfection with HIV-1 and HCV—A one-two punch. Gastroenterology.

[2] Chen J.Y., Feeney E.R., Chung R.T. (2014). HCV and HIV co-infection: Mechanisms and management. Nat. Rev. Gastroenterol. Hepatol..

[3] Hernandez M.D., Sherman K.E. (2011). HIV/HCV coinfection natural history and disease progression. Curr. Opin. HIV AIDS.

[4] Benhamou Y., Bochet M., di Martino V., Charlotte F., Azria F., Coutellier A., Vidaud M., Bricaire F., Opolon P., Katlama C. (1999). Liver fibrosis progression in human immunodeficiency virus and hepatitis C virus coinfected patients. Hepatology.

[5] Kovacs A., Al-Harthi L., Christensen S., Mack W., Cohen M., Landay A. (2008). CD8^+^ T cell activation in women coinfected with human immunodeficiency virus type 1 and hepatitis C virus. J. Infect. Dis..

[6] Justice A.C., Lasky E., McGinnis K.A., Skanderson M., Conigliano J., Fultz S.L., Crothers K., Rabeneck L., Rodriguez-Barradas M., Weissman S.B. (2006). Medical disease and alcohol use among veterans with human immunodeficiency infection: A comparison of disease measurement strategies. Med. Care.

[7] Hernandez-Gea V., Friedman S.L. (2011). Pathogenesis of accelerated fibrosis in HIV/HCV co-infection. Annu. Rev. Pathol. Mech. Dis..

[8] Schuppan D., Ruehl M., Somasundaran R., Hahn E.G. (2001). Matrix as modulator of stellate cell and hepatic fibrogenesis. Semin. Liver Dis..

[9] Bataller R., Gines P. (2002). New therapeutic strategies in liver fibrosis: Pathogenic basis. Med. Clin..

[10] Hui A., Friedman S.L. (2003). Molecular basis of hepatic fibrosis. Expert Rev. Mol. Med..

[11] Zhao Q., Qin C.Y., Zhao Z.H., Fan Y.C., Wang K. (2013). Epigenetic modifications in hepatic stellate cells contribute to liver fibrosis. Tohoku J. Exp. Med..

[12] Friedman S.L. (2008). Hepatic stellate cells: Protean, multifunctional, and enigmatic cells of the liver. Physiol. Rev..

[13] Friedman S.L. (2008). Mechanisms of hepatic fibrogenesis. Gastroenterology.

[14] Tsukamoto H. (2007). Epigenetic mechanism of stellate cell trans-differentiation. J. Hepatol..

[15] Rossmanith W., Schulte-Hermann R. (2001). Biology of transforming growth factor beta in hepatocarcinogenesis. Microsc. Res. Tech..

[16] Matsuzaki K. (2009). Modulation of TGF-β signaling during progression of chronic liver diseases. Front. Biosci..

[17] Bataller R., Brenner D.A. (2005). Liver fibrosis. J. Clin. Investig..

[18] Friedman S.L. (2000). Molecular regulation of hepatic fibrosis, an integrated cellular response to tissue injury. J. Biol. Chem..

[19] Zavadil J., Bottinger E.P. (2005). TGF-β and epithelial-to-mesenchymal transitions. Oncogene.

[20] Proell V., Carmona-Cuenca I., Murillo M.M., Huber H., Fabregat I., Mikulits W. (2007). TGF-β dependent regulation of oxygen radicals during transdifferentiation of activated hepatic stellate cells to myofibroblastoid cells. Comp. Hepatol..

[21] Seki E., de Minicis S., Osterreicher C.H., Kluwe J., Osawa Y., Brenner D.A., Schwabe R.F. (2007). TLR4 enhances TGF-β signaling and hepatic fibrosis. Nat. Med..

[22] Moreno M., Bataller R. (2008). Cytokines and renin-angiotensin system signaling in hepatic fibrosis. Clin. Liver Dis..

[23] Pinzani M., Gesualdo L., Sabbah G.M., Abboud H.E. (1989). Effects of platelet-derived growth factor and other polypeptide mitogens on DNA synthesis and growth of cultured rat liver fat-storing cells. J. Clin. Investig..

[24] Borkham-Kamphorst E., van Roeyen C.R., Ostendorf T., Floege J., Gressner A.M., Weiskirchen R. (2007). Pro-fibrogenic potential of PDGF-D in liver fibrosis. J. Hepatol..

[25] Pinzani M., Milani S., Herbst H., de Franco R., Grappone C., Gentilini A., Caligiuri A., Pellegrini G., Ngo D.V., Romanelli R.G. (1996). Expression of platelet-derived growth factor and its receptors in normal human liver and during active hepatic fibrogenesis. Am. J. Pathol..

[26] Wong L., Yamasaki G., Johnson R.J., Friedman S.L. (1994). Induction of β-platelet-derived growth factor receptor in rat hepatic lipocytes during cellular activation *in vivo* and in culture. J. Clin. Investig..

[27] Tsukamoto H., Zhu N.L., Asahina K., Mann D.A., Mann J. (2011). Epigenetic cell fate regulation of hepatic stellate cells. Hepatol. Res..

[28] Ramachandran P., Iredale J.P. (2012). Macrophages: Central regulators of hepatic fibrogenesis and fibrosis resolution. J. Hepatol..

[29] Guidotti L.G., Chisari F.V. (2006). Immunobiology and pathogenesis of viral hepatitis. Annu. Rev. Pathol..

[30] Negash A.A., Ramos H.J., Crochet N., Lau D.T., Doehle B., Papic N., Delker D.A., Jo J., Bertoletti A., Hagedorn C.H. (2013). IL-1β production through the NLRP3 inflammasome by hepatic macrophages links hepatitis C virus infection with liver inflammation and disease. PLoS Pathog..

[31] Durante-Mangoni E., Wang R., Crochet N., Lau D.T., Doehle B., Papic N., Delker D.A., Jo J., Bertoletti A., Hagedorn C.H. (2004). Hepatic CD1d expression in hepatitis C virus infection and recognition by resident proinflammatory CD1d-reactive T cells. J. Immunol..

[32] Hammam O., Mahmoud O., Zahran M., Aly S., Hosny K., Helmy A., Anas A. (2012). The role of Fas/Fas ligand system in the pathogenesis of liver cirrhosis and hepatocellular carcinoma. Hepat. Mon..

[33] Losikoff P.T., Self A.A., Gregory S.H. (2012). Dendritic cells, regulatory T cells and the pathogenesis of chronic hepatitis C. Virulence.

[34] Dolganiuc A., Norkina O., Kodys K., Catalano D., Bakis G., Marshall C., Mandrekar P., Szabo G. (2007). Viral and host factors induce macrophage activation and loss of Toll Like Receptor tolerance in chronic HCV infection. Gastroenterology.

[35] Coquillard G., Patterson B.K. (2009). Determination of hepatitis C virus-infected, monocyte lineage reservoirs in individuals with or without HIV coinfection. J. Infect. Dis..

[36] Gerlach J.T., Diepolder H.M., Jung M.C., Gruener N.H., Schraut W.W., Zachoval R., Hoffmann R., Schirren C.A., Santantonio T., Pape G.R. (1999). Recurrence of hepatitis C virus after loss of virus specific CD4^+^ T-cell response in acute hepatitis C. Gastroenterology.

[37] Lechner F., Wong D.K., Dunbar P.R., Chapman R., Chung R.T., Dohrenwend P., Robbins G., Phillips R., Klenerman P., Walker B.D. (2000). Analysis of successful immune responses in persons infected with hepatitis C virus. J. Exp. Med..

[38] Thimme R., Oldach D., Chang K.M., Steiger C., Ray S.C., Chisari F.V. (2001). Determinants of viral clearance and persistence during acute hepatitis C virus infection. J. Exp. Med..

[39] Harvey C.E., Post J.J., Palladinetti P., Freeman A.J., Ffrench R.A., Kumar R.K., Marinos G., Lloyd A.R. (2003). Expression of the chemokine IP-10 (CXCL10) by hepatocytes in chronic hepatitis C virus infection correlates with histological severity and lobular inflammation. J. Leukoc. Biol..

[40] Thio C.L., Gao X., Goedert J.J., Vlahov D., Nelson K.E., Hilgartner M.W., O’Brien S.J., Karacki P., Astemborski J., Carrington M. (2002). HLA-Cw*04 and hepatitis C virus persistence. J. Virol..

[41] McKiernan S.M., Hagan R., Curry M., McDonald G.S., Kelly A., Nolan N., Walsh A., Hegarty J., Lawlor E., Kelleher D. (2004). Distinct MHC class I and II alleles are associated with hepatitis C viral clearance, originating from a single source. Hepatology.

[42] Thursz M., Yallop R., Goldin R., Trepo C., Thomas H.C. (1999). Influence of MHC class II genotype on outcome of infection with hepatitis C virus. Lancet.

[43] Harris R.A., Sugimoto K., Kaplan D.E., Ikeda F., Kamoun M., Chang K.M. (2008). Human leukocyte antigen class II associations with hepatitis C virus clearance and virus-specific CD4 T cell response among Caucasians and African Americans. Hepatology.

[44] Apolinario A., Majano P.L., Alvarez-Pèrez E., Saez A., Lozano C., Vargas J., Garcìa-Monzòn C. (2002). Increased expression of T cell chemokines and their receptors in chronic hepatitis C: Relationship with the histological activity of liver disease. Am. J. Gastroenterol..

[45] Wang J., Holmes T.H., Cheung R., Greenberg H.B., He X.S. (2004). Expression of chemokine receptors on intrahepatic and peripheral lymphocytes in chronic hepatitis C infection: Its relationship to liver inflammation. J. Infect. Dis..

[46] Kusano F., Tanaka Y., Marumo F., Sato C. (2000). Expression of *C*–*C* chemokines is associated with portal and periportal inflammation in the liver of patients with chronic hepatitis C. Lab. Investig..

[47] Nishitsuji H., Funami K., Shimizu Y., Ujino S., Sugiyama K., Seya T., Takaku H., Shimotohno K. (2013). Hepatitis C virus infection induces inflammatory cytokines and chemokines mediated by the cross talk between hepatocytes and stellate cells. J. Virol..

[48] Dolganiuc A., Oak S., Kodys K., Golenbock D.T., Finberg R.W., Kurt-Jones E., Szabo G. (2004). Hepatitis C core and nonstructural 3 proteins trigger toll-like receptor 2-mediated pathways and inflammatory activation. Gastroenterology.

[49] Zeremski M., Petrovic L.M., Talal A.H. (2007). The role of chemokines as inflammatory mediators in chronic hepatitis C virus infection. J. Viral. Hepat..

[50] Wald O., Weiss I.D., Galun E., Peled A. (2007). Chemokines in hepatitis C virus infection: Pathogenesis, prognosis and therapeutics. Cytokine.

[51] Larrea E., Garcia N., Qian C., Civeira M.P., Prieto J. (1996). *Tumor necrosis factor α* gene expression and the response to interferon in chronic hepatitis C. Hepatology.

[52] Nelson D.R., Lim H.L., Marousis C.G., Fang J.W., Davis G.L., Shen L., Urdea M.S., Kolberg J.A., Lau J.Y. (1997). Activation of tumor necrosis factor-alpha system in chronic hepatitis C virus infection. Dig. Dis. Sci..

[53] Samuel V.T., Shulman G.I. (2012). Mechanisms for insulin resistance: Common threads and missing links. Cell.

[54] Khansari N., Shakiba Y., Mahmoudi M. (2009). Chronic inflammation and oxidative stress as a major cause of age-related diseases and cancer. Recent Pat. Inflamm. Allergy Drug Discov..

[55] Knolle P.A., Gerken G. (2000). Local control of the immune response in the liver. Immunol. Rev..

[56] Gao B., Jeong W.I., Tian Z. (2008). Liver: An organ with predominant innate immunity. Hepatology.

[57] Szabo G., Mandrekar P., Dolganiuc A. (2007). Innate immune response and hepatic inflammation. Semin. Liver Dis..

[58] Lin W., Tsai W.L., Shao R.X., Wu G., Peng L.F., Barlow L.L., Chung W.J., Zhang L., Zhao H., Jang J.Y. (2010). Hepatitis C virus regulates transforming growth factor beta1 production through the generation of reactive oxygen species in a nuclear factor κB-dependent manner. Gastroenterology.

[59] Lin W., Weinberg E.M., Tai A.W., Peng L.F., Brockman M.A., Kim K.A., Kim S.S., Borges C.B., Shao R.X., Chung R.T. (2008). HIV increases HCV replication in a TGF-β1-dependent manner. Gastroenterology.

[60] Presser L.D., Haskett A., Waris G. (2011). Hepatitis C virus-induced furin and thrombospondin-1 activate TGF-β1: Role of TGF-β1 in HCV replication. Virology.

[61] Shin J.Y., Hur W., Wang J.S., Jang J.W., Kim C.W., Bae S.H., Jang S.K., Yang S.H., Sung Y.C., Kwon O.J. (2005). HCV core protein promotes liver fibrogenesis via up-regulation of CTGF with TGF-β1. Exp. Mol. Med..

[62] Taniguchi H., Kato N., Otsuka M., Goto T., Yoshida H., Shiratori Y., Omata M. (2004). Hepatitis C virus core protein up-regulates transforming growth factor-beta 1 transcription. J. Med. Virol..

[63] Jaeschke H. (2002). Inflammation in response to hepatocellular apoptosis. Hepatology.

[64] Galle P.R., Hofmann W.J., Walczak H., Schaller H., Otto G., Stremmel W., Krammer P.H., Runkel L. (1995). Involvement of the CD95 (APO-1/Fas) receptor and ligand in liver damage. J. Exp. Med..

[65] Yoon J., Gores G. (2002). Death receptor-mediated apoptosis and the liver. J. Hepatol..

[66] Fischer R., Cariers A., Reinehr R., Häussinger D. (2002). Caspase 9-dependent killing of hepatic stellate cells by activated Kupffer cells. Gastroenterology.

[67] Kojima Y., Suzuki S., Tsuchiya Y., Konno H., Baba S., Nakamura S. (2003). Regulation of pro-inflammatory and anti-inflammatory cytokine responses by Kupffer cells in endotoxin-enhanced reperfusion injury after total hepatic ischemia. Transpl. Int..

[68] El Bassiouny A.E., El Bassiouni N.E., Nosseir M.M., Zoheiry M.M., El-Ahwany E.G., Salah F., Omran Z.S., Ibrahim R.A. (2008). Circulating and hepatic Fas expression in HCV-induced chronic liver disease and hepatocellular carcinoma. Medscape J. Med..

[69] Heydtmann M. (2009). Macrophages in hepatitis B and hepatitis C virus infections. J. Virol..

[70] Chen J.J., Sun Y., Nabel G.J. (1998). Regulation of the proinflammatory effects of Fas ligand (CD95L). Science.

[71] Canbay A., Feldstein A.E., Higuchi H., Werneburg N., Grambihler A., Bronk S.F., Gores G.J. (2003). Kupffer cell engulfment of apoptotic bodies stimulates death ligand and cytokine expression. Hepatology.

[72] Blackard J.T., Hiasa Y., Smeaton L., Jamieson D.J., Rodriguez I., Mayer K.H., Chung R.T. (2007). Compartmentalization of hepatitis C virus (HCV) during HCV/HIV coinfection. J. Infect. Dis..

[73] Blackard J.T., Sherman K.E. (2008). HCV/ HIV co-infection: Time to re-evaluate the role of HIV in the liver?. J. Viral. Hepat..

[74] Blackard J.T., Welge J.A., Taylor L.E., Mayer K.H., Kleir R.S., Celentano D.D., Jamieson D.J., Gardner L., Sheman K.E. (2011). HCV monoinfected, HIV monoinfected, HIV/HCV coinfected and HIV-seronegative/HCV-seronegative women. Clin. Infect. Dis..

[75] Han S.H., Kim S.U., Kim C.O., Jeong S.J., Park J.Y., Choi J.Y., Kim D.Y., Ahn S.H., Song Y.G. (2013). Abnormal liver stiffness assessed using transient elestography (Fibroscan) in HIV-infected patients without HBV/HCV coinfection receiving combined antiretroviral treatment. PLoS One.

[76] Hasson H., Merli M., Galli L., Gallotta G., Carbone A., Messina E., Bagaglio S., Morsica G., Salpietro S., Castagna A. (2013). Non-invasive fibrosis biomarkers—APRI and forms are associated with stiffness in HIV-monoinfected patients receiving antiretroviral drugs. Liver Int..

[77] Merchante N., Pérez-Chamacho I., Mira J.A., Rivero A., Macìas J., Camacho A., Gòmez-Mateos J., Garcìa-Làzaro M., Torre-Cisneros J., Pineda J.A. (2010). Prevalence and risk factors for abnormal liver stiffness in HIV-infected patients without viral hepatitis coinfection: Role of didanosine. Antivir. Ther..

[78] Kovari H., Ledergerber B., Battegay M., Rauch A., Hirschel B., Foguena A.K., Vernazza P., Bernasconi E., Mueller N.J., Webr R. (2010). Incidence and risk factors for chronic elevation of alanine aminotransferase levels in HIV-infected persons without hepatitis B or C virus co-infection. Clin. Infect. Dis..

[79] Bräu N., Salvatore M., Ríos-Bedoya C.F., Fernández-Carbia A., Paronetto F., Rodríguez-Orengo J.F., Rodríguez-Torres M. (2006). Slower fibrosis progression in HIV/HCV-coinfected patients with successful HIV suppression using antiretroviral therapy. J. Hepatol..

[80] Vlahakis S., Villasis-Keever A., Gomez T.S., Bren G.D., Paya C.V. (2003). Human immunodeficiency virus-induced apoptosis of human hepatocytes via CXCR4. J. Infect. Dis..

[81] Munshi N., Balasubramanian A., Koziel M., Ganjub R.K., Groopmanb J.E. (2003). Hepatitis C and human immunodeficiency virus envelope proteins cooperatively induce hepatocytic apoptosis via an innocent bystander mechanism. J. Infect. Dis..

[82] Balasubramanian A., Ganju R., Groopman J.E. (2003). HCV and HIV envelope proteins collaboratively mediate IL-8 secretion through activation of p38 MAP kinase and SHP2 in hepatocytes. J. Biol. Chem..

[83] Yoong K., Afford S.C., Jones R., Aujla P., Qin S., Price K., Hubscher S.G., Adams D.H. (1999). Expression and function of CXC and CC chemokines in human malignant liver tumors: A role for human monokine induced by g-interferon in lymphocyte recruitment to hepatocellular carcinoma. Hepatology.

[84] Wout A.B., Ran L.J., Kuiken C.L., Kootstra N.A., Pals S.T., Schuitemaker H. (1998). Analysis of the temporal relationchip between human immunodeficiency virus type 1 quasispecies in sequential blood samples and various organs obtained at autopsy. J. Virol..

[85] Tuyama A.C., Hong F., Saiman Y., Wang C., Ozkok D., Mosoian A., Chen P., Chen B.K., Klotman M.E., Bansal M.B. (2010). Human immunodeficiency virus (HIV)-1 infects human hepatic stellate cells and promotes collagen I and monocyte chemoattractant protein-1 expression: Implications for the pathogenesis of HIV/hepatitis C virus-induced liver fibrosis. Hepatology.

[86] Rotman Y., Liang T.J. (2009). Coinfection with hepatitis C virus and human immunodeficiency virus: Virological, immunological, and clinical out-comes. J. Virol..

[87] Lin W., Wu G., Li S., Weinberg E.M., Kumthip K., Peng L.F., Màndez-Navarro J., Chen W.C., Jilg N., Zhao H. (2011). HIV and HCV cooperatively promote hepatic fibrogenesis via induction of reactive oxygen species and NFκB. J. Biol. Chem..

[88] Bruno R., Galastri S., Sacchi P., Cima S., Caligiuri A., DeFranco R., Milani S., Gessani S., Fantuzzi L., Liotta F. (2010). gp 120 modulates the biology of human hepatic stellate cells: A link between HIV infection and liver fibrogenesis. Gut.

[89] Hong F., Tuyama A., Lee T.F., Loke J., Agarwal R., Cheng X., Garg A., Fiel M.I., Schwartz M., Walewski J. (2009). Hepatic stellate cells express functional CXCR4: Role in stromal cell-derived factor-1α-mediated stellate cell activation. Hepatology.

[90] Babu C.K., Suwansrinon K., Bren G.D., Badley A.D., Rizza S.A. (2009). HIV induces TRAIL sensitivity in hepatocytes. PLoS One.

[91] Tuyama A.C., Hong F., Schecter A.D., Mosoian A., Chen B.K., Chen P., Klotman M.E., Bansal M.B. (2007). HIV entry and replication in stellate cells promotes cellular activation and fibrogenesis: Implications for hepatic fibrosis in HIV/HCV coinfection. Hepatology.

[92] Schwabe R.F., Bataller R., Brenner D.A. (2003). Human hepatic stellate cells express CCR5 and RANTES to induce proliferation and migration. Am. J. Physiol. Gastrointest. Liver Physiol..

[93] Marra F., Valente A.J., Pinzani M., Abboud H.E. (1993). Cultured human liver fat-storing cells produce monocyte chemotactic protein-1. Regulation by proinflammatory cytokines. J. Clin. Investig..

[94] Efsen E., Bonacchi A., Pastacaldi S., Valente A.J., Wenzel U.O., Tosti-Guerra C., Pinzani M., Laffi G., Abboud H.E., Gentilini P. (2001). Agonist-specific regulation of monocyte chemoattractant protein-1 expression by cyclooxygenase metabolites in hepatic stellate cells. Hepatology.

[95] Marra F., de Franco R., Grappone C., Milani S., Pastacaldi S., Pinzani M., Romanelli R.G., Laffi G., Gentilini P. (1998). Increased expression of monocyte chemotactic protein-1 during active hepatic fibrogenesis: Correlation with monocyte infiltration. Am. J. Pathol..

[96] Balasubramanian A., Ganju R.K., Groopman J.E. (2006). Signal transducer and activator of transcription factor 1 mediates apoptosis induced by hepatitis C virus and HIV envelope proteins in hepatocytes. J. Infect. Dis..

[97] Matsuzaki K., Murata M., Yoshida K., Sekimoto G., Uemura Y., Sakaida N., Kaibori M., Kamiyama Y., Nishizawa M., Fujisawa J. (2007). Chronic inflammation associated with hepatitis C virus infection perturbs hepatic transforming growth factor β signaling, promoting cirrhosis and hepatocellular carcinoma. Hepatology.

[98] Seki E., de Minicis S., Gwak G.Y., Kluwe J., Inokuchi S., Bursill C.A., Llovet J.M., Brenner D.A., Schwabe R.F. (2009). CCR1 and CCR5 promote hepatic fibrosis in mice. J. Clin. Investig..

[99] Ochoa-Callejero L., Perez-Martinez L., Rubio-Mediavilla S., Oteo J.A., Martìnez A., Blanco J.R. (2013). Maraviroc, a CCR5 antagonist, prevents development of hepatocellular carcinoma in a mouse model. PLoS One.

[100] Affo S., Bataller R. (2011). RANTES antagonism: A promising approach to treat chronic liver diseases. J. Hepatol..

[101] Berres M.L., Koenen R.R., Rueland A., Zaldivar M.M., Heinrichs D., Sahin H., Schmitz P., Streetz K.L., Berg T., Gassler N. (2010). Antagonism of the chemokine Ccl5 ameliorates experimental liver fibrosis in mice. J. Clin. Investig..

[102] Dorr P., Westby M., Dobbs S., Griffin P., Irvine B., Macartney M., Mori J., Rickett G., Smith-Burchnell C., Napier C. (2005). Maraviroc (UK-427,857), a potent, orally bioavailable, and selective small-molecule inhibitor of chemokine receptor CCR5 with broad-spectrum anti-human immunodeficiency virus type 1 activity. Antimicrob. Agents Chemother..

[103] Fatkenheuer G., Pozniak A.L., Johnson M.A., Plettenberg A., Staszewski S., Hoepelman A.I., Saag M.S., Goebel F.D., Rockstroh J.K., Dezube B.J. (2005). Efficacy of short-term monotherapy with maraviroc, a new CCR5 antagonist, in patients infected with HIV-1. Nat. Med..

[104] Macias J., Viloria M.M., Rivero A., de los Santos I., Màrquez M., Postilla J., di Lello F., Camacho A., Sanz-Sanz J., Ojeda G. (2012). Lack of short-term increase in serum mediators of fibrogenesis and in non-invasive markers of liver fibrosis in HIV/hepatitis C virus-coinfected patients starting maraviroc-based antiretroviral therapy. Eur. J. Clin. Microbiol. Infect. Dis..

[105] Glässner A., Eisenhardt M., Kokordelis P., Krämer B., Wolter F., Nischalke H.D., Boesecke C., Sauerbruch T., Rockstroh J.K., Spengler U. (2013). Impaired CD4^+^ T cell stimulation of NK cell anti-fibrotic activity may contribute to accelerated liver fibrosis progression in HIV/HCV patients. J. Hepatol..

[106] Mehal W.Z., Friedman S.L. (2007). The Role of Inflammation and Immunity in the Pathogenesis of Liver Fibrosis. Liver Immunology.

[107] Gressner A.M., Weiskirchen R., Breitkopf K., Dooley S. (2002). Roles of TGF-β in hepatic fibrosis. Front. Biosci..

[108] Berenguer J., Fernandez-Rodriguez A., Jimenez-Sousa M.A., Cosìn J., Zarate P., Micheloud D., Lòpez J.C., Miralles P., Resino S. (2012). High plasma CXCL10 levels are associated with HCV-genotype 1, and higher insulin resistance, fibrosis, and HIV viral load in HIV/HCV coinfected patients. Cytokine.

[109] Sultana C., Erscoiu S.M., Grancea C., Ceausu E., Ruta S. (2013). Predictors of chronic hepatitis C evolution in HIV co-infected patients from Romania. Hepat. Mon..

[110] Cardin R., Saccoccio G., Masutti F., Bellentani S., Farinati F., Tiribelli C. (2001). DNA oxidative damage in leukocytes correlates with the severity of HCV-related liver disease: Validation in an open population study. J. Hepatol..

[111] Qu J., Zhang Q., Li Y., Liu W., Chen L., Zhu Y., Wu J. (2012). The Tat protein of human immunodeficiency virus-1 enhances HCV replication through interferon γ-inducible protein-10. BMC Immunol..

[112] Dinarello C.A. (1984). Interleukin-1 and the pathogenesis of the acute-phase response. N. Engl. J. Med..

[113] Allen I.C., Scull M.A., Moore C.B., Holl E.K., McElvania-TeKippe E., Taxman D.J., Guthrie E.H., Pickles R.J., Ting J.P. (2009). The NLRP3 inflammasome mediates *in vivo* innate immunity to influenza A virus through recognition of viral RNA. Immunity.

[114] Chakraborty S., Kaushik D.K., Gupta M., Basu A. (2010). Inflammasome signaling at the heart of central nervous system pathology. J. Neurosci. Res..

[115] Artlett C.M., Sassi-Gaha S., Rieger J.L., Boesteanu A.C., Feghali-Bostwick C.A., Katsikis P.D. (2011). The inflammasome activating caspase-1 mediates fibrosis and myofibroblast differentiation in systemic sclerosis. Arthritis Rheum..

[116] Daheshia M., Yao J.Q. (2008). The interleukin 1β pathway in the pathogenesis of osteoarthritis. J. Rheumatol..

[117] Dombrowski Y., Peric M., Koglin S., Kammerbauer C., Göss C., Anz D., Simanski M., Gläser R., Harder J., Hornung V. (2011). Cytosolic DNA triggers inflammasome activation in keratinocytes in psoriatic lesions. Sci. Transl. Med..

[118] Martinon F., Mayor A., Tschoppo J. (2009). The inflammasomes: Guardians of the body. Annu. Rev. Immunol..

[119] Kanneganti T.D. (2010). Central roles of NLRs and inflammasomes in viral infection. Nat. Rev. Immunol..

[120] Montserret R., Saint N., Vanbelle C., Salvay A.G., Simorre J.P., Ebel C., Sapay N., Renisio J.G., Böckmann A., Steinmann E. (2010). NMR structure and ion channel activity of the p7 protein from hepatitis C virus. J. Biol. Chem..

[121] Appay V., Sauce D. (2008). Immune activation and inflammation in HIV-1 infection: Causes and consequences. J. Pathol..

[122] Han D.W. (2002). Intestinal endotoxemia as a pathogenetic mechamism in liver failure. World J. Gastroenterol..

[123] De-Oca M.M., Marquez M., Soto M.J., Rodriguez-Ramos C., Terron A., Vergara A., Arizcorreta A., Fernandez-Gutierrez C., Giron-González J.A. (2011). Bacterial translocation in HIV-infected patients with HCV cirrhosis: Implications in hemodynamic alterations and mortality. J. Acquir. Immune Defic. Syndr..

[124] Guzman-Fulgencio M., Jimenez J.L., Berenguer J., Fernández-Rodríguez A., López J.C., Cosín J., Miralles P., Micheloud D., Muňoz-Fernández M.A., Resino S. (2012). Plasma IL-6 and IL-9 predict failure of interferon-α plus ribavirin therapy in HIV/HCV coinfected patients. J. Antimicrob. Chemother..

[125] Mattapallil J.J., Douek D.C., Hill B., Nishimura Y., Martin M., Roederer M. (2005). Massive infection and loss of memory CD4^+^ T cells in multiple tissues during acute SIV infection. Nature.

[126] Brenchley J.M., Price D.A., Schacker T.W., Asher T.E., Silvestri G., Rao S., Kazzaz Z., Bornstein E., Lambotte O., Altmann D. (2006). Microbial translocation is a cause of systemic immune activation in chronic HIV infection. Nat. Med..

[127] Landmann R., Knopf H.P., Link S., Sansano S., Schumann R., Zimmerli W. (1996). Human monocyte CD14 is upregulated by lipopolysaccharide. Infect. Immun..

[128] Sandler N.G., Koh C., Roque A., Eccleston J.L., Siegel R.B., Demino M., Kleiner D.E., Deeks S.G., Liang T.J., Heller T. (2011). Host response to translocated microbial products predicts outcomes of patients with HBV or HCV infection. Gastroenterology.

[129] Marchetti G., Cozzi-Lepri A., Merlini E., Bellistrì G.M., Castagna A., Galli M., Verucchi G., Antinori A., Costantini A., Giacometti A. (2011). Microbial translocation predicts disease progression of HIV-infected antiretroviral-naive patients with high CD4^+^ cell count. AIDS.

[130] Page E.E., Nelson M., Kelleher P. (2011). HIV and hepatitis C coinfection: Pathogenesis and microbial translocation. Curr. Opin. HIV AIDS.

[131] Balagopal A., Philp F.H., Astemborski J., Block T.M., Mehta A., Long R., Kirk G.D., Mehta S.H., Cox A.L., Thomas D.L. (2008). Human immunodeficiency virus-related microbial translocation and progression of hepatitis C. Gastroenterology.

[132] Henderson N.C., Iredale J.P. (2007). Liver fibrosis: Cellular mechanisms of progression and resolution. Clin. Sci..

[133] Godichaud S., Krisa S., Couronnè B., Dubuisson L., Mérillon J.M., Desmoulière A., Rosenbaum J. (2000). Deactivation of cultured human liver myofibroblasts by trans-resveratrol, a grapevine-derived polyphenol. Hepatology.

[134] Mastroianni C.M., Liuzzi M.G., D’Ettorre G., Lichtner M., Forcina G., di Campli N.F., Riccio P., Vullo V. (2002). Matrix metalloproteinase-9 and tissue inhibitors of matrix metalloproteinase-1 in plasma of patients co-infected with HCV and HIV. HIV Clin. Trials..

[135] Puoti M., Bonacini M., Spinetti A., Putzolu V., Govindarajan S., Zaltron S., Favret M., Callea F., Gargiulo F., Donato F. (2001). Liver fibrosis progression is related to CD4 cell depletion in patients coinfected with hepatitis C virus and human immunodeficiency virus. J. Infect. Dis..

[136] Mastroianni C.M., Liuzzi G.M. (2007). Matrix metalloproteinase dysregulation in HIV infection: Implications for therapeutic strategies. Trends Mol. Med..

[137] Eriksson S., Fraser J.R., Laurent T.C., Pertoft H., Smedsrød B. (1983). Endothelial cells are a site of uptake and degradation of hyaluronic acid in the liver. Exp. Cell. Res..

[138] Peters L., Mocroft A., Soriano V., Rockstroh J., Rauch A., Karlsson A., Knysz B., Pradier C., Zilmer K., Lundgren J.D. (2013). Hyaluronic acid levels predict risk of hepatic encephalopathy and liver-related death in HIV/viral hepatitis coinfected patients. PLoS One.

[139] Duong M., Petit J.M., Piroth L., Grappin M., Buisson M., Chavanet P., Hillon P., Portier H. (2001). Association between insulin resistance and hepatitis C virus chronic infection in HIV-hepatitis C virus-coinfected patients undergoing antiretroviral therapy. J. Acquir. Immune Defic. Syndr..

[140] Grigorescu M., Radu C., Crişan D., Grigorescu M.D., Şerban A., Neculoiu D., Rusu M., Acalovschi M. (2008). Metabolic syndrome, insulin resistance and adiponectin level in patients with chronic hepatitis C. J. Gastrointest. Liver Dis..

[141] Marra F., Bertolani C. (2009). Adipokines in liver diseases. Hepatology.

[142] Ryan P., Berenguer J., Michelaud D., Miralles P., Bellòn J.M., Alvarez E., Catàlan P., Sànchez-Conde M., Resino S. (2009). Insulin resistance is associated with advanced liver fibrosis and high body mass index in HIV/HCV-coinfected patients. J. Acquir. Immune Defic. Syndr..

[143] Carper M.J., Cade W.T., Cam M., Zhang S., Shalev A, Yarasheski K.E., Ramanadham S. (2008). HIV-protease inhibitors induce expression of suppressor of cytokine signaling-1 in insulin-sensitive tissues and promote insulin resistance and type 2 diabetes mellitus. Am. J. Physiol. Endocrinol. Metab..

[144] Capel E., Auclair M., Caron-Debarle M., Capeau J. (2012). Effect of ritonavir-boosted darunavir, atazanavir and lopinavir on adipose functions and insulin sensitivity in murine and human adipocyte. Antivir. Ther..

[145] Van Vonderen M.G., Blmer R.M., Hassink E.A., Sutinen J., Ackermans M.T., van Agtmael M.A., Yki-Jarvinen H., Danner S.A., Serlie M.J., Sauerwein H.P. (2010). Insulin sensitivity in multiple pathways is differently affected during zidovudine/lamivudine-containing compared with NRTI-sparing combination antiretroviral therapy. J. Acquir. Immune Defic. Syndr..

[146] Hruz PW. (2011). Molecular mechanisms for insulin resistance in treated HIV-infection. Best Pract. Res. Clin. Endocrinol. Metab..

[147] Hull M.W., Rollet K., Moodie E.E., Walmsley S., Cox J., Potter M., Cooper C., Pick N., Saeed S., Klein M.B. (2012). Insulin resistance is associated with progression to hepatic fibrosis in a cohort of HIV/hepatitis C virus-coinfected patients. AIDS.

[148] Browning J.D., Horton J.D. (2004). Molecular mediators of hepatic steatosis and liver injury. J. Clin. Investig..

[149] Hui J.M., Sud A., Farrell G.C., Bandara P., Byth K., Kench J.G., McCaughan G.W., George J. (2003). Insulin resistance is associated with chronic hepatitis C virus infection and fibrosis progression. Gastroenterology.

[150] Ratziu V., Munteanu M., Charlotte F., Bonyhay L., Poynard T. (2003). LIDO Study Group. Fibrogenic impact of high serum glucose in chronic hepatitis C. J. Hepatol..

[151] Hickman I.J., Powell E.E., Prins J.B., Clouston A.D., Ash S., Purdie D.M., Jonsson J.R.  (2003). In overweight patients with chronic hepatitis C, circulating insulin is associated with hepatic fibrosis: Implications for therapy. J. Hepatol..

[152] Paradis V., Perlemuter G., Bonvoust F., Dargere D., Parfait B., Vidaud M., Conti M., Huet S., Ba N., Buffet C. (2001). High glucose and hyperinsulinemia stimulate connective tissue growth factor expression: A potential mechanism involved in progression to fibrosis in nonalcoholic steatohepatitis. Hepatology.

[153] Grattagliano I., de Bari O., Bernardo T.C., Oliveira P.J., Portincasa P. (2012). Role of mitochondria in nonalcoholic fatty liver disease-from origin to propagation. Clin. Biochem..

[154] Perez-Matute P., Perez-Martinez L., Blanco J.R., Oteo J.A. (2013). Role of mitochondria in HIV infection and associated metabolic disorders: Focus on nonalcoholic fatty liver disease and lipodystrophy syndrome. Oxid. Med. Cell. Longev..

[155] Maagaard A., Holberg-Petersen M., Løvgården G., Holm M., Pettersen F.O., Kvale D. (2008). Distinct mechanisms for mitochondrial DNA loss in T and B lymphocytes from HIV-infected patients exposed to nucleoside reverse-transcriptase inhibitors and those naive to antiretroviral treatment. J. Infect. Dis..

[156] De Castro I.F., Berenguer J., Micheloud D., Guzmàn-Fulgencio M., Cosìn J., Alvarez E., Lòpez J.C., Miralles P., Garcìa-Alvarez M., Resino S. (2010). Serum levels of adipokines in HIV/HCV co-infected patients and their association with insulin resistance and liver disease severity. J. Infect..

[157] Tilg H., Moschen A.R. (2006). Adipocytokines: Mediators linking adipose tissue, inflammation and immunity. Nat. Rev. Immunol..

[158] Bertolani C., Marra F. (2008). The role of adipokines in liver fibrosis. Pathophysiology.

[159] Bertolani C., Sancho-Bru P., Failli P., Bataller R., Aleffi S., DeFranco R., Mazzinghi B., Romagnani P., Milani S., Ginès P. (2006). Resistin as an intrahepatic cytokine: Over-expression during chronic injury and induction of proinflammatory actions in hepatic stellate cells. Am. J. Pathol..

[160] Wang J., Leclercq I., Brymora J.M., Xu N., Ramezani-Moghadam M., London R.M., Brigstock D., George J. (2009). Kupffer cells mediate leptin-induced liver fibrosis. Gastroenterology.

[161] Pateria P., de Boer B., MacQuillan G. (2013). Liver abnormalities in drug and substance abuser. Best Pract. Res. Clin. Gastroenterol..

[162] Hézode C., Roudot-Thoraval F., Nguyan S., Grenard P., Julien B., Zafrani E.S., Pawlotsky J.M., Dhumeaux D., Lotersztajn S., Mallat A. (2005). Daily cannabis smoking as a risk factor for progression of fibrosis in chronic hepatitis C. Hepatology.

[163] Maurer H.H. (2010). Chemistry, pharmacology, and metabolism of emerging drugs of abuse. Ther. Drug Monit..

[164] Kothur R., Marsh F., Posner G. (1991). Liver function tests in nonparenteral cocaine user. Arch. Intern. Med..

[165] Silva M.O., Roth D., Reddy K.R., Fernandez J.A., Albores-Saavedra J., Schiff E.R. (1991). Hepatic dysfunction accompanying acute cocaine intoxication. J. Hepatol..

[166] Kielland K.B., Delaviris G.J., Rodge S., Eide T.J., Amundsen E.J., Dalgard O. (2014). Liver fibrosis progression at autopsy in injecting drug users infected by hepatitis C: A longitudinal long-term cohort study. J. Hepatol..

[167] Setshedi M., Wands J.R., Monte S.M. (2010). Acetaldehyde adducts in alcoholic liver disease. Oxid. Med. Cell. Longev..

[168] Farfán Labonne B.G., Gutiérrez M., Gómez-Quiroz L.E., Konigsberg Fainstein M., Bucio L., Souza V., Flores O., Ortíz V., Hernández E., Kershenobich D. (2009). Acetaldehyde-induced mitochondrial dysfunction sensitizes hepatocytes to oxidative damage. Cell Biol. Toxicol..

[169] Albano E., Vidali M. (2010). Immune mechanisms in alcoholic liver disease. Genes Nutr..

[170] Mello T., Ceni T., Surrenti C., Galli A. (2008). Alcohol induced hepatic fibrosis: Role of acetaldehyde. Mol. Asp. Med..

[171] Jeong W.I., Park O., Gao B. (2008). Abrogation of the antifibrotic effects of natural killer cells/interferon-γ contributes to alcohol acceleration of liver fibrosis. Gastroenterology.

[172] Rao R. (2009). Endotoxemia and gut barrier dysfunction in alcoholic liver disease. Hepatology.

